# Working memory deficits in children with schizophrenia and its mechanism, susceptibility genes, and improvement: A literature review

**DOI:** 10.3389/fpsyt.2022.899344

**Published:** 2022-08-05

**Authors:** Jintao Zhou, Jingfangzhou Li, Qi Zhao, Peixin Ou, Wan Zhao

**Affiliations:** ^1^School of Psychology, Nanjing Normal University, Nanjing, China; ^2^Department of Psychology, Fudan University, Shanghai, China; ^3^Department of Psychology, Faculty of Social Sciences, University of Macau, Macao, Macao SAR, China

**Keywords:** schizophrenia, children, working memory, genetic susceptibility, cognitive intervention

## Abstract

The negative influence on the cognitive ability of schizophrenia is one of the issues widely discussed in recent years. Working memory deficits are thought to be a core cognitive symptom of schizophrenia and lead to poorer social functions and worse academic performance. Previous studies have confirmed that working memory deficits tend to appear in the prodromal phase of schizophrenia. Therefore, considering that children with schizophrenia have better brain plasticity, it is critical to explore the development of their working memory. Although the research in this field developed gradually in recent years, few researchers have summarized these findings. The current study aims to review the recent studies from both behavior and neuroimaging aspects to summarize the working memory deficits of children with schizophrenia and to discuss the pathogenic factors such as genetic susceptibility. In addition, this study put forward some practicable interventions to improve cognitive symptoms of schizophrenia from psychological and neural perspectives.

## Introduction

Schizophrenia is now one of the most prevalent and serious mental illnesses in the world with typical symptoms defined (1, 2): (a) positive symptoms centered on delusions, hallucinations, disorganized speech, and grossly disorganized or catatonic behavior; (b) negative symptoms, i.e., affective flattening, alogia, or avolition; (c) cognitive symptoms including deficits in attention, working memory, and executive functions. Multiple psychiatrists proposed that different aspects of symptoms are not separate. For example, negative symptoms (loss of function), by definition, incorporate cognitive symptoms. However, more complex cognitive impairments have been found over the past years and viewed as one kind of independent symptom by multiple psychiatrists. As a result, we should treat schizophrenia symptoms from a unified perspective (3, 4). In addition, all these symptoms might be linked to the functions of dopamine, serotonin, and glutamatergic neurotransmission systems (5–7).

As shown in surveys, the lifetime prevalence of schizophrenia is estimated to be 0.3% worldwide and 0.83% in China (8, 9) and varies depending on the location, economic conditions, and the diagnostic criteria used. Although the annual cost of schizophrenia for both patients and society is tremendous (10), the therapeutic effect of schizophrenia is still not ideal. A previous investigation showed that only 13.5% of individuals with schizophrenia met the criteria for recovery (11). Furthermore, Phahladira and Luckhoff (12) have found that 70% of patients treated with antipsychotic medication achieved symptom remission, while only 9% achieved both functional remission and good quality of life, implying a large gap between symptom remission and a return to normal life. In a study by Nuechterlein, Subotnik (13), it was highlighted that cognitive functioning is one of the most critical determinants of quality of life for people with schizophrenia compared to symptoms such as delusions or hallucinations. More research provides sufficient support for this statement, for example, the important role of cognitive functioning in socialization has been demonstrated (14) and a longitudinal study has also shown that cognitive impairment predicts slower socialization (15). Therefore, cognitive functioning in patients with schizophrenia has become a key object of research.

People with schizophrenia have deficits in multiple cognitive domains simultaneously (1, 16), A meta-analysis has found that adult patients with schizophrenia perform more poorly than healthy controls in five cognitive domains including IQ, memory, language, executive function, and attention (17). Another systematic review has also indicated worse performance in a broad range of neuropsychological domains like multiple types of memory, intellectual function, sustained attention, as well as set-shifting and response inhibition (18). Specifically, working memory has been considered as one of the most fundamental cognitive functions and is related to a number of core symptoms of schizophrenia (19). Early studies demonstrated that there was a significant correlation between working memory deficits and several schizophrenia symptoms such as affective disorder and disorganized behavior (20, 21).

Working memory is defined as the limited capacity system necessary for short-term storage and manipulation of information in progress (22). In many studies investigating schizophrenia in all age groups, childhood and adolescence have been found to be particularly critical. Cognitive impairments such as working memory deficits tend to present in childhood (age 7–13) and adolescence (age 14–17) before they perform signs and symptoms of schizophrenia (23), so working memory deficits may be one of the predictors of schizophrenia in childhood. In addition, several empirical studies from schools have found that children in the early stages (the pre-psychotic prodromal period and the first 5–10 years after the initial episode) (24) of schizophrenia have poorer educational achievement and impaired social functioning, which may be due to working memory deficits (25–28). Remarkably, schizophrenia, one of the first-episode psychoses (FEP), often develops in childhood and adolescence (29), and patients with FEP tend to show a high remission rate owing to early intervention (30, 31), suggesting that the earlier the intervention was given, the more effective the intervention would be.

Therefore, it is meaningful to study schizophrenia in children from the perspective of cognitive impairments like working memory deficits, however, few reviews have been made. To conclude, the current study aims to summarize studies on working memory deficits in children with schizophrenia from both behavioral and neuroimaging aspects, try to explain deficits with possible susceptible genes and recommend some available interventions.

## Working memory deficits in children with schizophrenia

### Behavioral studies of working memory deficits in children with schizophrenia

Childhood and adolescence represent a rapid physical and psychological development. However, it is also a period when neurodevelopmental disorders such as schizophrenia begin to appear, accompanied with cognitive deficits (e.g., working memory deficits), (32). Cognitive Neuroscience Treatment Research to Improve Cognition in Schizophrenia (CNTRICS) has suggested four components of working memory deficits including goal maintenance, interference control, rehearsal (active maintenance over time), and updating (33). Previous studies have found deficits in goal maintenance among schizophrenia patients with several working memory tasks such as the anti-saccade task (34) and Dot Pattern Expectancy task (35). Some other studies also indicate impaired updating function in schizophrenia with updating and maintenance paradigm (36) and auditory target detection task (37). Eye-movement studies have also provided some evidence for abnormal working memory inhibitory processes in ultra-high-risk subjects and siblings (38, 39). These findings indicate that schizophrenia affects the working memory function of patients from multiple dimensions and we suggest that different mechanisms should be distinguished among these effects. In addition, some early field studies demonstrated possible working memory deficits in children with schizophrenia. A meta-analysis also showed that children with schizophrenia had worse general academic and mathematics achievements (40), meanwhile, accompanied by poorer social functions characterized by inadequate emotional reactions, the disorder of thinking, and loss of will (41, 42), which could be considered main markers of working memory deficits. Then, a questionnaire study on a large sample of adolescents and young adults has shown that specific psychotic-like experiences are associated with low working memory capacity (43). Furthermore, previous behavioral studies have found that children with schizophrenia tend to present more slow response time and low accuracy in different types of working memory tasks including visual, auditory, and verbal tasks (44–47). To conclude, sufficient behavioral evidence has told us that various working memory deficits exist in (high-risk) schizophrenia subjects and could be markers of the disease progression.

### Neuromechanism of working memory deficits in patients with schizophrenia

Over the past few years, an increasing number of neuroscientists have turned to explore the neuromechanism of working memory deficits in patients with schizophrenia. Many functional magnetic resonance imaging (fMRI) studies have reported altered engagement of the cerebral cortex during working memory processing in early-onset (EOS; the age of onset <18 years) schizophrenia, particularly in the dorsolateral prefrontal cortex (DLPFC), ventrolateral prefrontal cortex (VLPFC), and anterior cingulate cortex (ACC) (48–50). These regions have been shown to be closely associated with a wide range of executive dysfunction, especially memory deficits (51). For example, a previous study (52) found the decreased DLPFC connectivity within the working memory network and suggested that patients with EOS schizophrenia showed significant late developmental changes. Nielsen, Madsen (53) found that first-episode schizophrenia had decreased forward connectivity from the left inferior parietal lobe (IPL) to the left inferior frontal gyrus (IFG), which indicated the impaired working memory modulation of the frontoparietal network and the failure of context-sensitive coupling in the early phase of schizophrenia. Moreover, the degree of impact on different working memory functions varies, which perhaps means discrepant neuromechanism. A previous study found the increased activation when patients engaged in the manipulation plus maintenance task. Meanwhile, the changes of brain activation were disproportionately less in the DLPFC and greater in the VLPFC. This result suggested that manipulating function is selectively more affected than storing function in working memory (54). In addition, some researchers have found abnormal hyperactivation in the above-mentioned brain regions, reflecting the use of compensatory cognitive strategy while solving WM tasks (55), which might be another sign of working memory deficits. Furthermore, Loeb et al. (48) also pointed out that decreased activation and functional connectivity in the working memory network in childhood-onset schizophrenia were associated with the severity of the disease. To conclude, although there is no consensus on the neuromechanism of schizophrenia, it's clear that no matter in function or structure, numerous studies have offered enough evidence that children with or at risk of schizophrenia develop with apparent or potential working memory deficits and these deficits tend to present relatively early during schizophrenia.

## Genetic susceptibility factors of working memory deficits in schizophrenia patients

Previous studies provided sufficient evidence that schizophrenia is a highly heritable disease. An early twin study showed that the estimate of heritability in liability of schizophrenia was nearly 81% (56) and this result was still supported by some recent studies (57, 58). Similarly, working memory deficits in patients with psychosis are genetic with heritability estimates of up to 49% (59). Thus, numerous psychologists and psychiatrists have committed themselves to exploring possible susceptible genes linked with working memory deficits in patients with schizophrenia.

Previous genome-wide association studies (GWAS) have indicated some susceptibility genes are significantly associated with schizophrenia. The most compelling studies came from the psychiatric genomics Association (PGC), which provided the largest sample size for the GWAS study of schizophrenia. With the release of three PGC studies (60–62), researchers further clarified the genetic nature of schizophrenia. In 2014, the landmark GWAS study conducted by the PGC identified 128 independent single nucleotide polymorphisms (SNPs) in 108 gene loci that met genome-wide significance (61). Some associations at DRD2 and several genes involved in glutamatergic neurotransmission were consistent with previous leading pathophysiological hypotheses and highlight molecules with known and potential therapeutic relevance to schizophrenia. While 83 of 128 SNPs have not been reported previously, providing entirely new insights into etiology. In the recent GWAS study conducted by PGC, they identified 120 genes involved 106 protein-coding, of which more gene loci related to neurodevelopmental disorders were found (62). These GWAS studies have identified multiple susceptibility genes significantly related to schizophrenia. However, the specific mechanisms of these genes in schizophrenia are still unclear, which hinders the determination of promising drug molecular targets.

Some researchers chose to focus on the effects of schizophrenia susceptibility genes identified in the GWAS study on cognition and its brain mechanisms, many of which involved the working memory function. For example, the rs1344706 within the zinc finger protein 804A gene (*ZNF804A*) has been found a compelling candidate single nucleotide polymorphism (SNP) for schizophrenia (63). Recent studies have also provided support for altered brain activities among *ZNF804A* variant rs1344706 schizophrenia carriers, such as influenced hippocampal-DLPFC functional connectivity during resting-state (64), abnormal prefrontal connectivity (65), and altered cortical network dynamics (66). Some of them also play a significant role in working memory performance and a previous cognitive-measuring study has indicated higher polygenic scores of *ZNF804A* were associated with poorer working memory (67). Linden, Lancaster (68) has found worse working memory performance for face processing among *ZNF804A* carriers. Additionally, impaired cognitive control function in working memory has also been found (69, 70).

The dopamine receptor type 2 gene (*DRD2*) polymorphism was also known as one of the risk variants for schizophrenia (71). The polymorphism of *DRD2* (C957T) has been known to be associated with schizophrenia (72), while the result seems inconsistent in different populations (73, 74). Meanwhile, the C957T variant has showed closely connected to working memory performance, especially in goal maintenance function (75) but limited effect on executive function (Working Memory, Response Inhibition, Cognitive Flexibility) (76). Moreover, researchers have also found that the rs1076560 variant of *DRD2* significantly contributes to abnormal prefrontal activity and impaired working memory in several task-related studies (77, 78). Braun, Harneit (79) have studied the role of *DRD2* dopamine in whole-brain network dynamics and found decreased stability of working memory representations. Pergola, Di Carlo (80) comprehensively studied the *DRD2* co-expression network and calculated a related polygenic index which could significantly predict the abnormal prefrontal activity and insufficient working memory processing.

Several GWAS studies have also identified the *CACNA1C* gene polymorphism (rs1006737) as a susceptibility locus for schizophrenia (81) which often comes with alterations in prefrontal activation and fronto-hippocampal connectivity during emotional processing and executive cognition (82, 83) and finally lead to poor working memory performance (84, 85), especially impact on the encoding phase of working memory (86). Beyond that, some other SNPs in *CACNA1C* have been identified as risk loci for schizophrenia over the past years (87). However, we are still lacking in profound understanding among them and more evidence is expected.

Moreover, *RELN* variants have been proved to contribute to the endophenotypes of schizophrenia (88) and regulate synaptic plasticity and cognition in psychotic subjects (89). Reproducible studies have shown multiple *RELN* variants (7q21-32) are associated with memory impairments (90, 91). In addition, a study in healthy population carried with *RELN* (rs362691) has shown high cognitive deficits, especially in executive function (92). Although more and more SNPs in *RELN* are confirmed to be related to schizophrenia recently (93), their effect on working memory awaits deeper studies.

In addition, the transcription factor 4 gene (*TCF4*) (94, 95), the microRNA-137 gene (MIR-137) (96–98), and the type-3 metabotropic glutamate receptor gene (*GRM3*) have also been found to be associated with working memory impairment by affecting the functions of brain regions related to working memory processes, including DLPFC, hippocampus, striatum and other regions.

Beyond that, Nicodemus, Kolachana (99) proposed that statistical interactions among multiple susceptibility genes should also be considered. The polygenic risk score (PRS) has become a powerful indicator of GWAS results. Identified SNPs were weighted by their association odds ratio to construct a risk score for each individual. The PRS was positively correlated with the genetic susceptibility of the disease. Wang, Liu (100) calculated the PRS using the formula developed by PGC and found that a higher PRS was significantly associated with impairment in working memory. To sum up, a large number of GWAS studies have shown us that schizophrenia susceptible genes indeed account for working memory deficits. These findings will undoubtedly provide enlightenment for improved treatment for cognitive symptoms in schizophrenia patients in the future. In addition, many newly identified genes are related to neurodevelopmental disorders (62), which further suggests that we need to pay more attention to the children with schizophrenia.

## Intervention with cognition deficits in children with schizophrenia

It has been commonly accepted that children, compared to adults, develop with better neural and behavioral plasticity, so early intervention for children with schizophrenia is necessary. In addition, although the main treatment of schizophrenia is antipsychotics, some other therapies have also been put forward to promote the recovery of cognitive function in schizophrenia patients, which in turn help establish adaptive behaviors and have gradually been introduced to children.

Cognitive behavioral therapy (CBT) refers to a class of interventions that share the basic premise that mental disorders and psychological distress are maintained by cognitive factors (101, 102). Therefore, CBT approaches are often used in responding to some cognitive symptoms in a wide range of psychiatric disorders and show a large effect size (103). A review of meta-analysis on the efficacy of CBT has confirmed that the preferential application of CBT in children helped to alleviate behavioral and emotional disorders in various psychosis to some extent (104). In the meantime, a randomized controlled study (105) has found that CBT with treatment as usual (9 months) contributed to better global function and quality of life in adolescents with early-onset schizophrenia, which is considered the external sign of cognitive recovery. Another study on the CBT for adolescents at high risk for serious mental illness (SMI) such as schizophrenia and bipolar disorder indicated that patients asked to engage in several interventions showed reduced difficulties in emotion regulation, better frustration tolerance, and stronger motivation (106). More importantly, CBT is proved to be highly acceptable and feasible with few side effects (104, 107).

Another similar psychopharmacologic treatment commonly used for mental disorders is mindfulness. The definitions of mindfulness are various and have been divided into five dimensions including awareness and attention, present-centeredness, external events, cultivation, and present centeredness (108). An early meta-analysis showed that mindfulness intervention was moderately effective in treating psychotic negative symptoms (109). Recent meta-analyses have provided more evidence for its efficacy and safety (110, 111). Furthermore, mindfulness among children and adolescents has been suggested to have certain clinical effects, while it still needs to be standardized (112). Therefore, Mindfulness-based cognitive therapy for children (MBCT-C) was developed (113) and significant effects were found, including improved cognitive control, better emotional control, and fewer behavioral symptoms (114–116). More importantly mindfulness-based approaches were highly feasible among children and adolescents (117). However, there are few studies on the efficacy of MBCT-C for children, especially schizophrenia. More clinical studies are expected and a comparison could be made between CBT and mindfulness.

However, the tremendous time and human cost of CBT and mindfulness cannot be ignored. To solve this issue, many researchers have been dedicated to intervention with specific cortexes via several neuro-technologies, such as electroconvulsive therapy (ECT). ECT has been proved to be well-tolerated, well-established, and efficacious in multiple moods and psychiatric disorders including depression and schizophrenia (118, 119). However, the majority of results from older studies in children and adolescents were mixed and its effect on them could not be clearly explained. Nowadays, studies and applications of ECT among children and adolescents with FEP become popular and its efficacy has got some verification (120, 121), while more clinic researches are still required. In addition, the side effect of ECT, particularly in cognitive function (e.g., memory loss), has been in heavy discussion for a long time (122, 123). Dossing and Pagsberg (124) suggested that more attention should be paid to cognitive dysfunction under ECT in further studies. To conclude, ECT has been considered a reliable way especially for antipsychotics-resistance patients, while the effect on cognitive functions remains to be vague.

To avoid the argument about safety and enhance the feasibility of neurotechnology, several non-invasive brain stimulation techniques were developed. Transcranial magnetic stimulation (TMS) uses very brief high intensity magnetic fields to induce currents and thus depolarize neurons in small regions of the cortex (125). A review showed that TMS has been applied for the treatment of auditory verbal hallucinations in schizophrenia patients and proved superior efficacy (126). Another repetitive TMS study in EOS patients (127) also reported a significant improvement in the severity of auditory hallucinations and global functioning over the left temporoparietal junction. Additionally, the author appealed to concern about the TMS used in the treatment of several cognitive functions, especially among children. Related studies have shown that TMS in specific cortex like DLPFC contributed to the improvement of inhibitory and executive function in children and early adults (128, 129). However, recent meta-analyses indicate that cognitive enhancement effects caused by TMS in patients with schizophrenia or other neuropsychiatric conditions are moderate (130, 131) and researchers attribute the result to insufficient subjects and lack of long-term TMS intervention. Therefore, more studies in this field are desperately needed.

Another non-invasive brain stimulation technique is transcranial direct current stimulation (tDCS), which has been the subject of great interest owing to its easiness of operation and relatively low cost. tDCS takes effect by applying a weak current over the scalp to modulate cortical excitability by facilitating or inhibiting ongoing neuronal processes. A review of tDCS in psychiatric disorders has demonstrated its high tolerability in patients with schizophrenia and improvement in positive and negative symptoms (132). Meanwhile, one study has provided support that tDCS also played a role in the treatment of cognitive impairments, especially in working memory (133). On this basis, some other researchers focus on the feasibility and safety of tDCS among children and adolescents which is proved to be well-tolerated (134, 135). A recent study of tDCS among children and adolescents with attention-deficit/hyperactivity disorder (ADHD) have indicated positive result in attention and inhibitory control function (136). Another study has reported enhancing working memory performance in children with ADHD under the intervention of tDCS over the left DLPFC (137). In addition, improved reward processing in children with ADHD has also been found through tDCS in specific regions including the left DLPFC and the right ventromedial prefrontal cortex (138). However, a meta-analysis showed little evidence that 1 to 5 sessions of tDCS improve the cognitive measure of children with ADHD (139), which calls for a persistent tDCS study in psychopathic children. Moreover, the study on the clinical effects of tDCS among children with schizophrenia is limited and its real efficacy with cognitive impairments remains to be further examined.

Above all, the current treatments of children with schizophrenia were focused on antipsychotics, which could greatly improve some positive symptoms such as hallucinations and paranoia. In addition, ECT could further enhance the efficiency of treatment in conjunction. However, the above- mentioned therapies are not very effective for negative symptoms (e.g., avolition) and cognitive symptoms (e.g., working memory deficits). Therefore, it is highly recommended to use TMS and tDCS. Moreover, psychopharmacologic treatment including CBT and mindfulness play an important role in helping children with schizophrenia improve cognitive functions and adapt to daily life. Moreover, it is worth emphasizing that persistent treatment is necessary no matter what kind of therapy is chosen.

## Conclusion

The current review mainly discusses the cognitive impairments in patients with schizophrenia, especially in children. Previous studies have confirmed that children with schizophrenia are prone to show worse academic grades and more conflicting relationships, accompanied by poor performance in several working memory tasks. In addition, numerous neuroimaging studies have demonstrated altered activation of working memory networks and weak functional connectivity in schizophrenia children. To further explain the pathogenesis of schizophrenia, many susceptible genes have been put forward to take effect in related activities among specific cerebral cortexes which finally accounts for working memory deficits in schizophrenia. Thus, findings of the early presence of cognitive symptoms both in behavior and cerebral cortex offer the opportunity for prevention and treatment of schizophrenia in children. Currently, lots of psychologists and psychiatrists have contributed to the enhancement of cognitive impairments of children with schizophrenia by employing CBT, mindfulness, ECT, TMS, and tDCS.

Although studies on working memory deficits among children with schizophrenia have been constantly emerging, several limitations still exist. Firstly, most working memory tasks used in experiments are mainly designed for adults, and their difficulty and duration might be unsuitable for children. Thus, adapted working memory tasks are required to assess multiple working memory functions more efficiently. Similarly, the treatment for children with schizophrenia is nearly the same as that of adults, and there is also a lack of child intervention studies. Considering the better brain plasticity of children, the brain activity of children might be different from that of adults, so more specific treatment should be studied and developed. Furthermore, the intervention duration of several existing studies on children is not enough which may be one of the reasons for the inconsistent treatment effects. Therefore, more long-term follow-up studies are needed.

In the future, the mechanism of working memory and other cognition impairments in schizophrenia are needed to be explored. On this basis, it is worth looking forward to the more effective and lower-cost therapy for schizophrenia patients, especially for children.

## Author contributions

WZ had full access to all the information in this review. WZ, JZ, and JL managed the literature searches and selection. WZ and QZ reviewed the logicality of the manuscript. PO and QZ polished the manuscript in language expression. JZ wrote the first draft of the manuscript. All authors contributed to the article and approved the submitted version.

## Conflict of interest

The authors declare that the research was conducted in the absence of any commercial or financial relationships that could be construed as a potential conflict of interest.

## Publisher's note

All claims expressed in this article are solely those of the authors and do not necessarily represent those of their affiliated organizations, or those of the publisher, the editors and the reviewers. Any product that may be evaluated in this article, or claim that may be made by its manufacturer, is not guaranteed or endorsed by the publisher.
